# Travel-associated Diseases, Indian Ocean Islands, 1997–2010

**DOI:** 10.3201/eid1908.121739

**Published:** 2013-08

**Authors:** Hélène Savini, Philippe Gautret, Jean Gaudart, Vanessa Field, Francesco Castelli, Rogelio López-Vélez, Poh Lian Lim, Marc Shaw, Frank von Sonnenburg, Louis Loutan, Fabrice Simon, for the GeoSentinel Surveillance Network

**Affiliations:** Laveran Military Teaching Hospital, Marseille, France (H. Savini, F. Simon);; University Hospital Institute Méditerranée Infection, Marseille (P. Gautret);; Aix Marseille University, Marseille (J. Gaudart); University College London, London, UK (J. Gaudart);; InterHealth, London (V. Field); National Travel Health Network and Centre, London (V. Field);; University of Brescia, Brescia, Italy (F. Castelli);; Ramón y Cajal Hospital, Madrid, Spain (R. López-Vélez);; Tan Tock Seng Hospital, Singapore (P.L. Lim);; Worldwise Travellers Health Center, Auckland, New Zealand (M. Shaw);; Ludwig Maximilian University of Munich, Munich, Germany (F. von Sonnenburg);; Geneva University Hospital, Geneva, Switzerland (L. Loutan);; University of Geneva, Geneva (L. Loutan)

**Keywords:** travelers, Indian Ocean, Comoros, Madagascar, Seychelles, Mauritius, Maldives, Réunion Island, vector-borne infections, parasites, viruses, diarrhea, malaria, dengue, chikungunya, arboviruses, infectious diseases

## Abstract

Data collected by the GeoSentinel Surveillance Network for 1,415 ill travelers returning from Indian Ocean islands during 1997–2010 were analyzed. Malaria (from Comoros and Madagascar), acute nonparasitic diarrhea, and parasitoses were the most frequently diagnosed infectious diseases. An increase in arboviral diseases reflected the 2005 outbreak of chikungunya fever.

The outbreak of chikungunya fever in Indian Ocean islands (IOI) provides new insights on emerging infections in this geographic region (*1*). We present data collected over 14 years from travelers to IOI who visited GeoSentinel clinics.

## The Study

GeoSentinel sites are specialized travel clinics providing surveillance data for ill travelers. Detailed methods for recruitment of patients for the GeoSentinel database are described elsewhere (*2*). Demographics, travel characteristics, and individual medical data were obtained from travelers to Comoros (including Mayotte), Madagascar, Maldives, Mauritius, Réunion Island, and Seychelles during March 1, 1997–December 31, 2010. Statistical significance was determined by using Fisher exact test for categorical variables and Kruskal-Wallis test for quantitative variables. A 2-sided significance level of p<0.05 was considered significant.

This study comprised 1,415 ill patients (Table 1). Demographic data varied according to the visited island. Median age was 36 years, and the male to female ratio was 1.1:1.0. The most common reason for travel was tourism (44.5%), followed by visiting friends and relatives (VFR) (30.8%). Only 43.0% of travelers had a pre-travel encounter with a travel medicine specialist or general practitioner.

**Table 1:** Characteristics of 1,415 ill travelers returning from Indian Ocean islands, 1997–2010*

Characteristic	All islands, n = 1,415	Madagascar, n = 502	Comoros, n = 444	Maldives, n = 174	Mauritius, n = 153	Seychelles, n = 81	Réunion Island, n = 39	>1 Island, n = 22
Female sex, %†	47.8	47.2	42.3	51.7	51.6	63	53.8	50
Median age, y (95% CI)†	36 (19–65)	33 (20–66)	39 (18–64)	34 (6–62)	37 (15–69)	39 (24–69)	33 (14–65)	32 (23–62)
Median travel duration, d (95% CI)†	29 (7–341)	30 (8–665)	41 (12–176)	14 (5–366)	14 (6–109)	14 (7–112)	19 (2–3270)	35 (9–416)
Reason for travel, %†								
Tourism	44.5	53.8	5.6	62.6	81.0	85.2	48.7	59.1
VFR	30.8	5.2	89.4	0	5.2	1.2	10.3	0.0
Missionary/volunteer/ student/military	12.9	24.7	1.4	21.9	3.3	1.2	5.1	27.3
Business	10.9	15.9	1.8	15.5	10.5	11.1	30.8	9.1
Other	0.1	0.4	1.8	0	0	1.2	5.1	4.5
Pre-travel health advice, %†	43.3	55.2	32.2	47.1	35.9	37	25.6	72.7
Inpatient care, %	30.0	7.2	79.3	8.0	9.2	3.7	12.8	4.5

*VFR, visiting friends and relatives. †p<0.01 for the comparison among islands.

**Figure 1:**
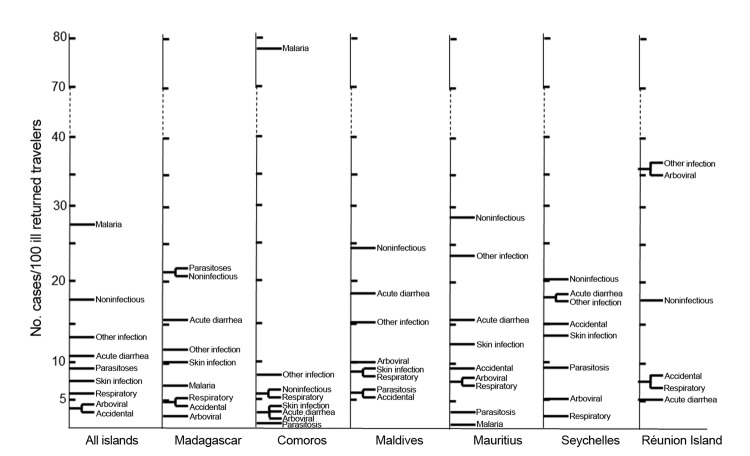
Relative proportion of different diagnoses among 1,415 ill travelers returning from Indian Ocean islands, 1997–2010. The numbers are shown for each diagnosis for all ill travelers returning from each island. Some patients had >1 diagnosis. Malaria: *Plasmodium falciparum* infection (341 cases, including 12 severe cases), *P. vivax* infection (24), *P. ovale* infection (11), *P. malariae* infection (10). Acute diarrheal infections: campylobacteriosis (12), salmonellosis (6), shigellosis (5). Parasitic infections: gardiasis (33), schistosomiasis (21), strongyloidiasis (13), miyases (13), amoebiasis (9), cutaneous larva migrans (9), trichuriasis (7), ascariasis (5), hookworm infection (5), enterobiasis (2), neurocysticercosis (2), filariasis (1), blastocystosis (1). Respiratory infections: upper respiratory tract infections (26), influenza (6), tuberculosis (4). Arboviral infections: chikungunya (40), dengue (24). Other infections: urinary tract infections (22), leptospirosis (2), rickettsial infections (3), Q fever (1). Among accidental diseases: insect bites (28), rabies postexposure treatments (6), marine envenomization (5).

Illness patterns varied by place of exposure (Figure 1). Malaria, the most frequently diagnosed illness (388 [27.4%] travelers), accounted for 74.1% of diagnoses for VFR but only 6.6% for non-VFR travelers (p<0.01). *Plasmodium falciparum* malaria represented 88.0% of cases, including 12 cases of severe malaria, mostly from Comoros or Madagascar. One case of *P. ovale* malaria was reported from Mauritius in a person who had previously traveled to Cameroon.

**Figure 2:**
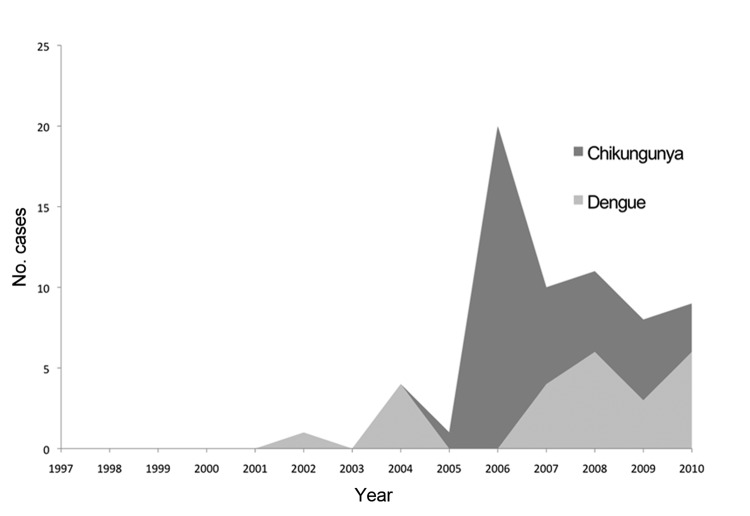
Annual occurrence of arboviral disease cases (dengue and chikungunya) among 1,415 travelers returning from Indian Ocean islands and seen at GeoSentinel sites, 1997–2010.

Arboviral disease diagnoses included 40 cases of chikungunya and 24 cases of dengue. Overall, arboviral diseases accounted for 4.5% of the total diagnoses. Arboviral diseases accounted for 36.0% of diseases acquired by travelers to Réunion Island (vs. 3.6% in non–Réunion Island travelers, p<0.01) and were more frequent in tourists than in nontourists (6.5% vs. 2.9%, p<0.01). Numbers of arboviral diseases showed a sustained increase and peaked in 2006. Dengue was noted only after 2001. Chikungunya cases dramatically increased in 2006 and were sustained at a lower level during 2007–2010, suggesting local transformation from epidemic to endemic phases or better notification of the diagnosis (Figure 2).

Parasitic infections other than malaria accounted for 131 (9.3%) diagnoses. A higher proportion of parasitoses occurred in travelers to Madagascar than in persons who had not traveled there (21.3% vs. 2.6%, p<0.01) and in missionary than non-missionary travelers (18.7% vs. 7.9%, p<0.01). Intestinal helminths or protozoans were the most commonly identified parasites. Schistosomiasis (21 cases) was reported from Madagascar only.

Acute nonparasitic diarrhea accounted for 162 (11.5%) final diagnoses. Higher proportions of such diarrhea occurred in travelers to Madagascar than in persons who had not traveled there (15.7% vs. 9.1%, p<0.01) and in travelers to Maldives than in persons who had not traveled there (18.4% vs. 10.5%, p<0.01). In 23 (14.2%) cases, a pathogen was identified. Acute nonparasitic diarrhea and skin infections were more frequently reported in tourists than in nontourists (17.3% and 12.4% vs. 6.8% and 3.8%, respectively [p<0.01]). The proportion of respiratory infections was higher in persons traveling for business than in persons traveling for other reasons (11.2% vs. 5.1%, p<0.01).

Mosquito bites, food and water consumption, and direct contact with skin were the most frequent modes of disease transmission (Table 2). The proportion of mosquito-transmitted diseases was higher among travelers to Comoros than among other travelers (80.2% vs. 10.0%, p = 0.006). The proportion of foodborne diseases was higher among travelers to Madagascar than in travelers to other areas (27.5% vs. 10.9%, p<0.001) and to Maldives than to other areas (23.0% vs. 15.8%, p = 0.03). Diseases transmitted through skin contact accounted for a higher proportion of diagnoses in travelers returning from Madagascar than from other areas (18.1% vs. 7.6%, p<0.001). Compared with nonbusiness travelers, business travelers had a higher proportion of respiratory-transmitted diseases (1.9% vs. 12.3%, p<0.001) and sexually and blood-transmitted diseases (0.3% vs. 6.6%, p = 0.03).

**Table 2:** Modes of disease transmission for 1,415 ill travelers returning from Indian Ocean islands, 1997–2010

Mode of transmission	Total no. (%), n = 1,415	Island visited, no. (%) travelers						
		Madagascar, n = 502	Comoros, n = 444	Maldives, n = 174	Mauritius, n = 153	Seychelles, n = 81	Réunion Island, n = 39	>1 Island, n = 22
Mosquito bite	452 (31.9)	48 (9.6)	356 (80.2)	17 (9.8)	13 (8.5)	4 (4.9)	14 (35.9)	0 (0.0)
Food/water consumption	236 (16.7)	138 (27.5)	10 (2.3)	40 (23.0)	27 (17.6)	20 (24.7)	3 (7.7)	2 (9.1)
Direct skin contact	159 (11.2)	91 (18.1)	9 (2.0)	20 (11.5)	24 (15.7)	17 (21.0)	0	2 (9.1)
Respiratory droplet	102 (7.2)	33 (6.6)	25 (5.6)	20 (11.5)	15 (9.8)	7 (8.6)	8 (20.5)	4 (18.2)
Animal contact	44 (3.1)	15 (3.0)	0	7 (4.0)	10 (6.5)	12 (14.8)	1 (2.6)	1 (4.5)
Fresh water contact	23 (1.6)	21 (4.2)	0	0	1 (0.7)	0	1 (2.6)	0
Sex/blood	7 (0.5)	3 (0.6)	1 (0.2)	1 (0.6)	0	1 (1.2)	1 (2.6)	0
Tick bite	3 (0.2)	1 (0.2)	1 (0.2)	0	1 (0.7)	0	0	0

## Conclusions

This large study addresses travel-associated diseases in travelers returning from IOI. *P. falciparum* infection was the most common reason for seeking post-travel care, notably when returning from Comoros, a well-known malaria-endemic archipelago (*3*). Imported malaria is frequently described in France, particularly in Marseille, which is the preferred residence city for migrants from Comoros and their descendants (*4*). Previous reports have shown that VFR sought pre-travel advice less frequently than did other travelers, possibly because of economic concerns, language barriers, or cultural beliefs (*5*–*7*). We observed a lower proportion of malaria in persons who had traveled to Madagascar, where both *P. falciparum* and *P. vivax* are endemic, and only 1 case in a traveler to Mauritius, where few cases are reported (*3*). No malaria cases were identified from Réunion Island, Seychelles, or Maldives, which is consistent with travel medicine guidelines that do not recommend chemoprophylaxis for travelers visiting these islands (*8*).

The reports of dengue and chikungunya fever from all islands reflect the wide distribution of the vector, *Aedes* spp. mosquitoes. Our results parallel those of the chikungunya fever outbreak that spread throughout IOI during 2005–2006 (*9*), facilitated by an adaptive virus mutation that led to increased infectivity, replication, and transmission by *A. albopictus* mosquitoes (*10*). The outbreak affected hundreds of travelers to IOI (*11*). Concern about the possible spread of chikungunya fever increased with the autochthonous outbreak of chikungunya fever in Italy in 2007 that developed from a patient returning from India (*12*). This sporadic case confirmed the ability of the virus to settle in countries colonized by *Aedes* sp. mosquitoes as a result of increasing intercontinental exchanges. Surveillance of travelers with a view toward early diagnosis is a key element in controlling outbreaks of imported arboviral diseases.

Parasitic infections, including schistosomiasis, accounted for a major proportion of final diagnoses in travelers to Madagascar, where these infections represent a public health concern (*13*). Testing for such diseases should be considered in ill travelers returning from this island.

Nonparasitic diarrhea was reported mainly in tourists returning from Madagascar and the Maldives. Few pathogens were documented, reflecting the practice of empiric antimicrobial treatment before laboratory testing (*14*). The higher incidence of diarrheal illness among tourists could be explained by an immature mucosal immunity (*15*) and easier access to medical care.

Business travelers had a higher proportion of respiratory diseases, independent of the island visited. This finding may relate to longer stays in air conditioned hotels and close human-to-human contact in this population.

These data have at least 4 limitations. First, we included only returning travelers who were ill and receiving care at GeoSentinel sites. Second, self-limited diseases or diseases of short duration may be underrepresented. Third, the lack of a denominator does not permit calculation of prevalence. Fourth, diseases with very short or very long incubation periods might not, with certainty, be attributed to any particular destination. Nevertheless, our study describes the spectrum of diseases among travelers returning from each IOI based on robust numbers of ill travelers.

Ill travelers returning from IOI are heterogeneous in their demographic and travel characteristics and display specific diseases that depend on the island and the travel reason. These findings reflect the different economic, ecologic, and public health situations found across this region (Technical Appendix Table). More than two thirds of diseases in travelers to IOI were, theoretically, preventable by reinforcing food and hand hygiene and by avoiding insect bites or direct contact with soil and fresh water. Most travelers in our survey traveled to a single island; thus, targeted destination-specific pre-travel advice and post-travel medical management of ill persons should be provided on a country-level basis rather than addressed nonspecifically.

Technical Appendix Geographic, political, economic, and health characteristics of Indian Ocean islands, 1997–2010.
