# Supplementary material for: Travel-associated Diseases, Indian Ocean Islands, 1997–2010
Source: Emerg Infect Dis. 2013 Aug;19(8):1297–301. doi: 10.3201/eid1908.121739 (PMC3739505; doi:10.3201/eid1908.121739)
Supplement: Technical Appendix — Geographic, political, economic, and health characteristics of Indian Ocean islands, 1997–2010. [file 12-1739-Techapp-s1.pdf]

Article DOI: http://dx.doi.org/10.3201/eid1908.121739

Travel-associated Diseases, Indian Ocean Islands, 1997–2010

Technical Appendix

Technical Appendix Table. Geographic, political, economic, and health characteristics of Indian Ocean islands, 1997–2010*

	Indian Ocean Islands						
	Comoros archipelago						
Characteristic	Comoros	Mayotte	Madagascar	Réunion Island	Mauritius	Seychelles	Maldives
Population	676,000	186,500	19,625,000	825,000	1,288,000	84,000	309,000
Area, km^2	1,862	374	592,000	2,512	18,600	453	298,000
Climate	Tropical	Tropical	Tropical	Tropical	Tropical	Tropical	Tropical
Temperature range, °C	23–27	24–28	10–37	18–31	17–30	21–31	26–34
Political status	Independent, 1973	French collectivity	Independent, 1956	French oversea department	Independent, 1968	Independent, 1976	Independent, 1968
Tourists arrivals, no./y	18,000	40,670	229,000	409,000	761,000	129,000	683,000*
Gross national income, $/inhabitants	1,170	4578	1,050	7,752	12,580	19,650	5,290
Life expectancy at birth, M/F, y	58/62	73/76	63/67	75/82	69/76	69/77	74/76
Expenditure on health, % GDP	3.4	NA	4.1	NA	5.7	4.0	8.0
Access to sanitation, rural/urban, %	15–38	100–100	27–49	100–100	98–100	100–100	98–100
Leading 3 causes of death, all ages	Malaria, perinatal, diarrhea	Heart, tumors, traumatic	Respiratory infections, malaria, diarrhea	Heart, tumors, trauma	Heart, cerebrovascular, diabetes mellitus	Hypertension, heart, respiratory infections	Heart, respiratory infections, perinatal

*NA, data not available. Sources: www. afro.who.int 2006; www.outre-mer.gouv.fr; www.weather.com; www.who.int/countries 2009; www.diplomatie.gouv.fr; Tourism year book 2009, Ministry of Tourism, Arts and Culture, Republic of Maldives.
